# Rapid Differentiation of Human Embryonic Stem Cells into Testosterone-Producing Leydig Cell-Like Cells *In vitro*

**DOI:** 10.1007/s13770-021-00359-8

**Published:** 2021-06-24

**Authors:** Eun-Young Shin, Seah Park, Won Yun Choi, Dong Ryul Lee

**Affiliations:** 1 Department of Biomedical Science, CHA University, 335 Pangyo-ro, Bundang-gu, Seongnam-si, 13488 Gyeonggi-do Republic of Korea; 2 Fertility Center, CHA Ilsan Woman’s & Children’s General Hospital, 1205 Jungang-ro, Ilsandong-gu, Goyang-si, 10414 Gyeonggi-do Republic of Korea

**Keywords:** Leydig cells, Human embryonic stem cells, Differentiation, Testosterone, Hypogonadism

## Abstract

**Background:**

Leydig cells (LCs) are testicular somatic cells that are the major producers of testosterone in males. Testosterone is essential for male physiology and reproduction. Reduced testosterone levels lead to hypogonadism and are associated with diverse pathologies, such as neuronal dysfunction, cardiovascular disease, and metabolic syndrome. LC transplantation is a promising therapy for hypogonadism; however, the number of LCs in the testis is very rare and they do not proliferate *in vitro*. Therefore, there is a need for an alternative source of LCs.

**Methods:**

To develop a safer, simple, and rapid strategy to generate human LC-like cells (LLCs) from stem cells, we first performed preliminary tests under different conditions for the induction of LLCs from human CD34/CD73 double positive-testis-derived stem cells (HTSCs). Based on the embryological sequence of events, we suggested a 3-step strategy for the differentiation of human ESCs into LLCs. We generated the mesendoderm in the first stage and intermediate mesoderm (IM) in the second stage and optimized the conditions for differentiation of IM into LLCs by comparing the secreted testosterone levels of each group.

**Results:**

HTSCs and human embryonic stem cells can be directly differentiated into LLCs by defined molecular compounds within a short period. Human ESC-derived LLCs can secrete testosterone and express steroidogenic markers.

**Conclusion:**

We developed a rapid and efficient protocol for the production of LLCs from stem cells using defined molecular compounds. These findings provide a new therapeutic cell source for male hypogonadism.

## Introduction

Male hypogonadism is characterized by diminished functional activity in the testes, which may result in reduced production of androgens. This can be caused by genetic defects or acquired factors, such as trauma, infection, drug use, chemotherapy, radiation, and aging [1]. Furthermore, male hypogonadism, commonly characterized by low serum testosterone levels, can lead to mood disturbance, decreased bone mineral density, amyotrophy, sexual dysfunctions, adiposity, increased cardiovascular disease, and diabetes [2, 3]. These phenomena are well known as symptoms of “andropause,” “late-onset hypogonadism,” and “testosterone deficiency syndrome.” Testosterone replacement therapy (TRT) is commonly used for male hypogonadism, but it has adverse effects, such as heart attack, stroke, and death [4]. Furthermore, exogenous testosterone administration suppresses the feedback loop for endogenous testosterone production, which is not suitable for long-term therapy [1]. Therefore, finding an alternative therapy that is safer than TRT and can be used long-term is needed.

Recently, Leydig cell (LC) transplantation has been considered as an alternative approach to overcome male hypogonadism [5–7]. LCs are testicular somatic cells found in the testis interstitium that produce testosterone, accounting for approximately 95% of circulatory testosterone [8, 9]. Testosterone synthesis in LCs is modulated by the hypothalamic–pituitary–gonadal (HPG) axis [10]. LCs have luteinizing hormone and choriogonadotropin receptor (LHCGR) on the plasma membrane, which can be stimulated by LH from the pituitary gland. When LH binds the LHCGR of LCs, LH activates adenylate cyclase, which can lead to an increase in intracellular cyclic adenosine monophosphate (cAMP) production [11]. cAMP activates protein kinase A (PKA), which promotes the transfer of cholesterol from the extracellular lipoprotein and cholesterol pool to the inner mitochondrial membrane of LCs [12]. This process is mediated by steroidogenic acute regulatory protein (STAR), which is a cholesterol transfer protein on the mitochondrial membrane [13]. Delivered cholesterol is metabolized into pregnenolone by the cytochrome P450 cholesterol side-chain cleavage enzyme (CYP11a1). Pregnenolone moves to the smooth endoplasmic reticulum (sER) and is metabolized to testosterone through stepwise reactions mediated by enzymes, such as cytochrome P450 17α-hydroxylase (CYP17a1), 17β-hydroxysteroid dehydrogenase (17βHSD), and 3β-hydroxysteroid dehydrogenase (3βHSD) [14]. The expression levels of these enzymes are closely related to the ability of LCs to produce testosterone [15, 16].

LCs can be divided into four types based on their differential stages: stem LCs (SLCs), progenitor LCs (PLCs), immature LCs (iLCs), and adult LCs (ALCs). SLCs are undifferentiated mesenchymal-like stem cells characterized by their ability to self-renew and differentiate into multilineage cells and LCs [5]. Previously, our group reported that CD34/CD73-double positive-testis-derived stem cells (HTSCs) have a higher proliferative potential than bone marrow mesenchymal stem cells [17]. Furthermore, HTSCs have the ability for multipotent differentiation into chondrogenic, osteogenic, adipogenic, pancreatic, and neuronal lineages. These characteristics are similar to those of SLCs; therefore, we assumed that HTSCs might differentiate into LCs.

Recently, several studies have demonstrated that SLC transplantation is a promising therapy for male hypogonadism [5, 18, 19]. However, it has limitations in clinical use because of the lack of specific markers for isolation, the rare proportion of adult testes, and the necessity for supporting factors from other testicular somatic cells, such as Sertoli and peritubular myoid cells, that induce differentiation of SLCs into functional ALCs. In particular, these cells are relatively rare or damaged in males with hypogonadism; thus, it is difficult to obtain SLCs and induce differentiation into ALCs *in vivo*. Additionally, SLCs for autologous transplantation must be isolated before damages, such as aging, chemotherapy, and radiation. Therefore, an alternative therapeutic method is needed for further clinical applications. Previous studies demonstrated that stem cell-derived LLCs may be promising cells for the therapy of testosterone deficiency [20–28]. However, most studies have induced differentiation through genetic modifications, such as ectopic expression of steroidogenic factor 1 (SF1), which may be unsafe for clinical applications. Chen et al. developed a small-molecule-based strategy without ectopic expression for producing LLCs from induced pluripotent stem cells (iPSCs), but it still had some drawbacks. Their method required a long period (approximately a month) and complicated recipes for inducing LLCs [27].

**Fig. 1:**
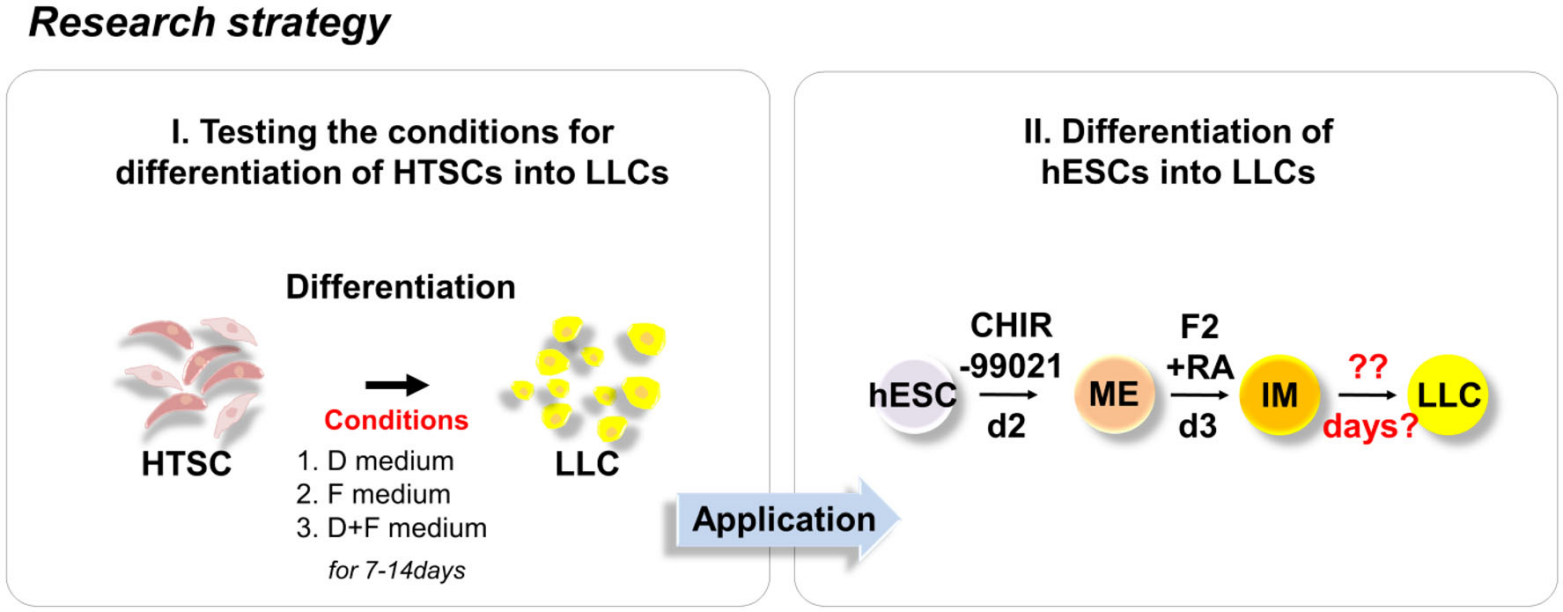
Schematic illustration of the strategy for the generation of LLCs from hESCs. HTSCs, human CD34/CD73 double positive-testis-derived stem cells; LLCs, Leydig cell-like cells; hESCs, human embryonic stem cells; ME, mesendoderm; IM, intermediate mesoderm; F2, FGF2; RA, retinoic acid

Therefore, we aimed to develop a safer, simpler, and rapid strategy for the differentiation of human embryonic stem cells (hESCs) into LLCs using molecular compounds. To achieve this purpose, we used HTSCs that might harbor SLC properties for optimization of the conditions for LC differentiation because SLCs are known as progenitors of LCs in neonatal testis (Fig. 1) [29]. We found a few defined molecular compounds that could directly differentiate hESCs into LLCs for a short period. Furthermore, these hESC-derived LLCs can successively produce testosterone *in vitro*. Our findings may serve as a rapid and efficient strategy to obtain functional LLCs for *in vitro* experimental disease modeling, drug screening, toxicity testing, and replacement therapies for male hypogonadism.

## Materials and methods

### Cell culture

All experiments were performed following the guidelines of the Institutional Review Board for Human Research at CHA Gangnam Medical Center (06-06 and GCI-14-16), Seoul, South Korea for research using hESCs and HTSCs. Isolation of HTSCs from testis and cultivation of HTSCs were previously described [17] and obtained from CHA Gangnam Medical Center, Seoul, South Korea. HTSCs were cultured in a 1:1 mixture of Dulbecco's modified Eagle’s medium: nutrient mixture F12 (DMEM/F12): StemPro®- 34 SFM supplemented with 10% fetal bovine serum (FBS) and 1% penicillin/streptomycin solution (P/S; all from Gibco, Grand Island, NY, USA).

The human embryonic stem cell (hESC) line, CHA-hES15 (Korea Stem Cell Registry No. hES12010028), was cultured on a mouse embryonic fibroblast (MEFs) feeder layer. hESCs were maintained in DMEM/F12 supplemented with 20% KnockOut serum replacement (kSR), 1% non-essential amino acids (NEAA), 0.1% β-mercaptoethanol, and 4 ng/mL human recombinant basic fibroblast growth factor (bFGF; all from Gibco-BRL).

### Isolation of testicular cells (TCs)

Isolated testicular cells (iTCs) were obtained as previously described [30]. To isolate the iTCs, testis tissues were obtained from patients undergoing testicular sperm extraction (TESE) at the CHA Gangnam Medical Center, Seoul, South Korea. Testis tissues were washed in Dulbecco’s phosphate-buffered saline (PBS, Hyclone, GE Healthcare Life Sciences, Marlborough, MA, USA) and then digested for 90–120 min with gentle agitation at 37 °C in 200 mL of DMEM/F12 containing an enzyme mixture (0.1% collagenase type IV + 10 µg/mL DNase + 1 µg/mL soybean trypsin inhibitor; all from Gibco-BRL). After digestion, the cells were filtered through a 70 µm mesh. Cells were then washed twice with DMEM/F12 medium and resuspended in DMEM/F12 supplemented with 10% FBS + ITS (Insulin-transferrin-selenium; Invitrogen, Grand Island, NY, USA). After 2 h, floating cells were removed and adherent cells, which were iTCs, were cultured for use as a positive control for analysis.

### Cell differentiation

Undifferentiated human ESCs were seeded onto Geltrex (Gibco-BRL)-coated plates in ES cell culture medium (DMEM/F12 supplemented with 20% kSR + 1% NEAA + 0.1% β-mercaptoethanol + 4 ng/mL bFGF; all from Gibco-BRL). After 24 h later, to induce mesendoderm (ME) differentiation, the cells were treated with Advanced-RPMI 1640 (A-RPMI 1640) supplemented with 1% L-GultaMAX (L-glu, Gibco-BRL) + 1% P/S + 5 µM CHIR99021 (Glycogen synthase kinase-3 inhibitor; Stemgent, Lexington, MA) for 2 days. For intermediate mesoderm (IM), cells at the ME stage were treated with A-RPMI 1640 supplemented with 1% L-glu + 1% P/S + 100 ng/mL FGF2 (Peprotech, Rocky Hill, NJ, USA) + 1 µM retinoic acid (RA, Sigma, St. Louis, MO, USA) for 3 days.

For LLC differentiation, cells were cultured in the basal medium (phenol red-free DMEM/F12 supplemented with 2% FBS + ITS) in all experiments. To induce LLCs from HTSCs, cells were incubated in D medium (previously reported in [31], 10 ng/mL platelet-derived growth factor beta, PDGF-BB; Peprotech + 1 ng/mL luteinizing hormone, LH; Sigma + 1 nM 3,3,5-triiodo-l-thyronine sodium salt, thyroid hormone; Sigma + 70 ng/mL Insulin-like growth factor-1, IGF-I; Peprotech), F medium (100 µM forskolin) and D + F medium (10 ng/mL PDGF-BB + 1 ng/mL LH + 1 nM thyroid hormone + 70 ng/mL IGF-I + 100 µM forskolin) were used. To induce the LLCs from hESCs, cells at the IM stage were treated with D + F medium (10 ng/mL PDGF-BB + 1 ng/mL LH + 1 nM thyroid hormone + 70 ng/mL IGF-I + 100 µM forskolin) or D + F + F2 medium (10 ng/mL PDGF-BB + 1 ng/mL LH + 1 nM thyroid hormone + 70 ng/mL IGF-I + 100 µM forskolin + 10 ng/mL FGF2) for 4 days. The D + F > F2 group was treated with D + F medium for 4 days and then with 10 ng/mL FGF2 for 3 days.

### RNA extraction and RT-PCR

Total RNA was isolated from cells and tissues using TRIzol reagent (Invitrogen) according to the manufacturer’s instructions. The concentration of RNA was quantified using a NanoDrop 2000 Spectrophotometer (Thermo Scientific, Waltham, MA, USA). RNA was reverse transcribed using the First Strand cDNA Synthesis kit (Takara Bio, Shiga, Japan) according to the manufacturer’s protocol. cDNA was amplified using the AccuPower PCR premix (Bioneer, Daejeon, Korea), and all PCR products were separated by 2% agarose gel electrophoresis and observed under ultraviolet illumination. The following primers were used for RT-PCR: *T* forward 5′-CTCACAGACCACAGGCTGG-3′; *T* reverse 5′-TTTATCCATGCTGCAATCCC-3′; *GSC* forward 5′-CAGCTGGCCCGGAAAGTGCACCTC-3′; *GSC* reverse 5′-TTCTCCGGTGACGCCTTCGACGAC-3′; *MIXL1* forward 5′-TTGGTTCAAAGCTGGACTCA-3′; *MIXL1* reverse 5′-CTGTCAGTCATGGCTCCTCA-3′; *PAX2* forward 5′-CAAAGTTCAGCAGCCTTTCC-3′; *PAX*2 reverse 5′-CCACACCACTCTGGGAATCT-3′; *OSR1* forward 5′-TAGTTGGTGAGCTGCAGGG-3′; *OSR1* reverse 5′-TTCAGCTAAAGCCCCAGAGA-3′; *LHX1* forward 5′-ATCCTGGACCGCTTTCTCTT-3′; *LHX1* reverse 5′-GTACCGAAACACCGGAAGAA-3′; *GATA4* forward 5′-CTGTGCCCGTAGTGAGATGA-3′; *GATA4* reverse 5′-TCCAAACCAGAAAACGGAAG-3′; *WT1* forward 5′-GGGTACGAGAGCGATAACCA-3′; *WT1* reverse 5′-TCTCACCAGTGTGCTTCCTG-3′; *SF1* forward 5′-CCCTGCTTGACTACACCCTG-3′; *SF1* reverse 5′-AATAACCACATCCTGGTGAAAGA-3′; *STAR* forward 5′-TAGCGACATTCAAGCTGTGC-3′; *STAR* reverse 5′-GGTTAATCCACGTGCTAGGG-3′; *LIFR* forward 5′-CTCCATGTGTGGGACATTCA-3′; *LIFR* reverse 5′-CCGTTCTTGTTATCAGTTGGAGA-3′; *LHCGR* forward 5′-GGCCGGTCTCACTCGAC-3′; *LHCGR* reverse 5′-GAGGTTGTCAAAGGCATTAGC-3′; *3βHSD* forward 5′-GCCTGTTGGTGGAAGAGAAG-3′; *3βHSD* reverse 5′-GGCTCATCCAGAATGTCTCC-3′; *PDGFRα* forward 5′-CTCCATGTGTGGGACATTCA-3′; *PDGFRα* reverse 5′-AGCTGGCAGAGGATTAGGCT-3′; *CYP11a1* forward 5′-TCAGTGATGACCTGTTCCGC-3′; *CYP11a1* reverse 5′-ATCAATGAATCGCTGGGCCT-3′; *CYP17a1* forward 5′-ATTCGGTTCGTATGGGCACC-3′; *CYP17a1* reverse 5′-GTTGTTGGACGCGATGTCTA-3′; *17βHSD* forward 5′-CCAAAGTCTTTCTTGCGGTC-3′; *17βHSD* reverse 5′-TGGCCTCTAGTTTTTCCAGC-3′; *GAPDH* forward 5′-AGAAGGCTGGGGCTCATT-3′; *GAPDH* reverse 5′-AGGGGCCATCCACAGTCT-3′.

### Immunocytochemistry

Cells were washed with PBS and then fixed with 4% paraformaldehyde (Biosesang, Gyeonggi-do, Korea) for 20 min at room temperature (RT). Fixed cells were permeabilized with 0.1% Triton X-100 (Sigma) in PBS for 10 min. After permeabilization, the cells were washed twice with PBS, blocked with blocking solution (DAKO North America Inc., Carpinteria, CA, USA) for 1 h at RT, and incubated with primary antibodies overnight at 4 °C. On day 2, cells were washed twice with PBS, incubated with secondary antibodies for 1 h at RT, and then analyzed. The following antibodies were used: SF1 (1:100; LS-C162999, Lifespan Biosciences, Inc., Seattle, WA, USA), GATA4 (1:100; sc-25310, Santa Cruz Biotechnology, Santa Cruz, CA, USA); 3βHSD (1:50; sc-30820, Santa Cruz), and LHCGR (1:100; ab204950, Abcam, Cambridge, MA, USA). The secondary antibodies used were Alexa Fluor 488-conjugated anti-rabbit IgG (1:200, Invitrogen, Carlsbad, CA, USA) for SF1, Alexa Fluor 555-conjugated anti-mouse IgG (1:200, Invitrogen) for GATA4 and LHCGR, and Alexa Fluor 555-conjugated anti-goat IgG (1:100, Invitrogen) for 3βHSD.

### Testosterone measurement by enzyme-linked immunosorbent assay (ELISA)

Cell culture supernatants were collected at each differentiation endpoint for the quantitative measurement of testosterone. Cells were subcultured for 48 h before harvest and equally seeded on Geltex-coated plates. The concentration of testosterone in the medium was measured using testosterone ELISA kits (582,701, Cayman Chemical Co., Ann Arbor, MI, USA) according to the manufacturer’s instructions.

## Results

### Characterization of HTSCs

**Fig. 2:**
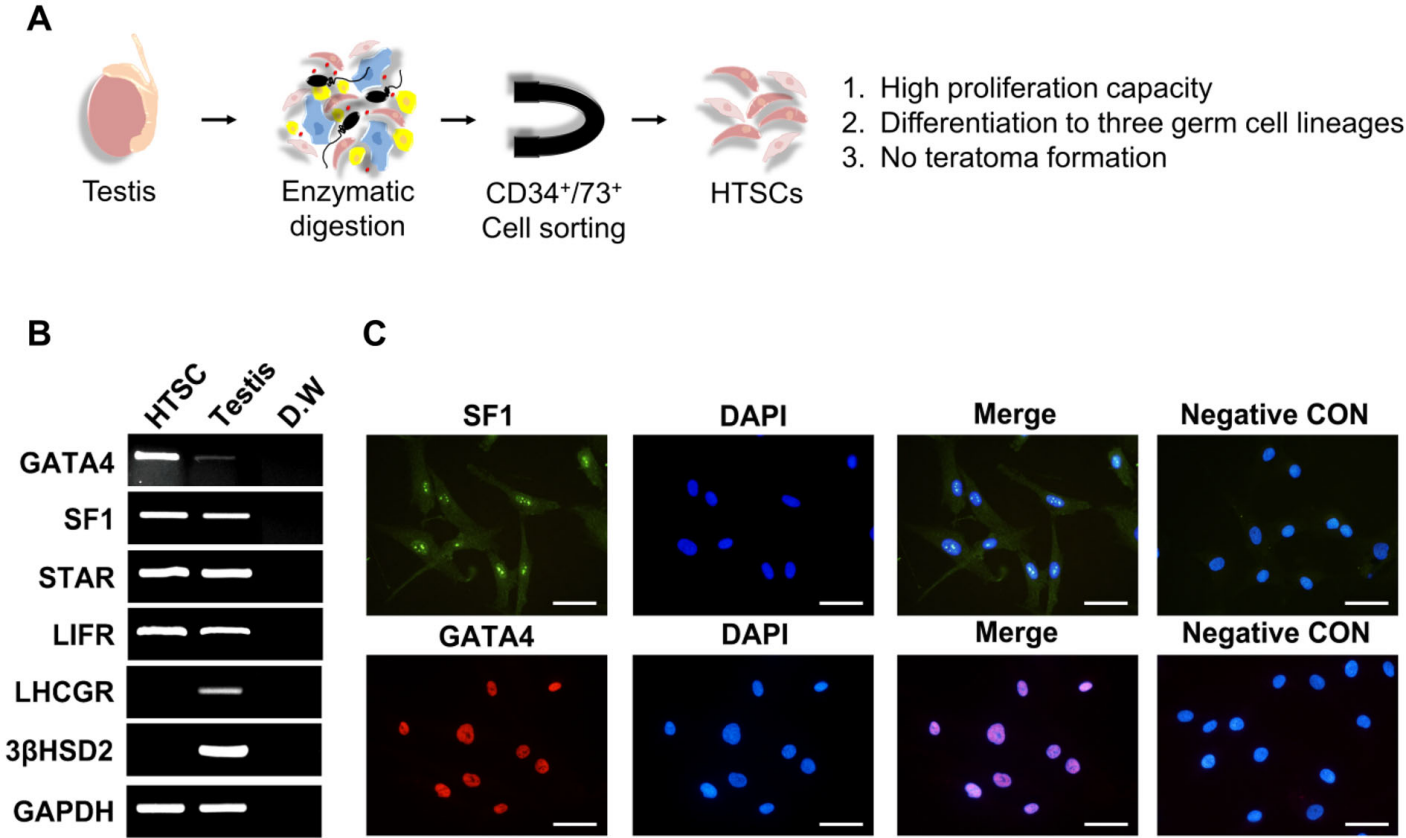
Characterization of HTSCs. **A** Schematic illustration for isolation of HTSCs.** B** Expression of genes encoding transcription factors in LC development (GATA4), stem LCs (LIFR), steroidogenic cells (SF1 and STAR), and mature adult LCs (LHCGR and 3βHSD) in HTSCs.** C** Immunofluorescence staining of the steroidogenic cell marker (SF1) and key transcription factor for LC (GATA4) in HTSCs. Scale bars = 50 μm. HTSCs, highly proliferative testis-derived stem cells; LCs, Leydig cells; GATA4, GATA-binding protein 4; SF1, steroidogenic factor 1; STAR, steroidogenic acute regulatory protein; LIFR, leukemia inhibitory factor receptor; LHCGR, luteinizing hormone and choriogonadotropin receptor; 3βHSD, 3β-hydroxysteroid dehydrogenase; GAPDH, glyceraldehyde 3-phosphate dehydrogenase

Our previous study showed that CD34/CD73 double-positive cells in the testis have high proliferation capacity and can differentiate into three germ layer lineages, without forming teratomas (Fig. 2A) [17]. These characteristics are comparable with those of SLCs; thus, we investigated whether HTSCs have SLC properties by assessing the gene expression of SLC markers in isolated HTSCs. Human SLCs are positive for SF1, GATA-binding protein 4 (GATA4), STAR, and leukemia inhibitory factor receptor (LIFR) but negative for ALC markers, such as LHCGR and 3βHSD [18, 19, 32]. Consistent with this, HTSCs were positive for the SLC lineage marker (LIFR), a key transcription factor for LC development (GATA4), and steroidogenic markers (SF1 and STAR) but were negative for ALC markers (LHCGR and 3βHSD) (Fig. 2B). Additionally, similar to the PCR results, the protein expression of SF1 and GATA4 was confirmed by immunostaining, and both proteins were well expressed in HTSCs (Fig. 2C). These results indicate that HTSCs have SLC properties.

### HTSCs can differentiate into LLCs *in vitro*

**Fig. 3:**
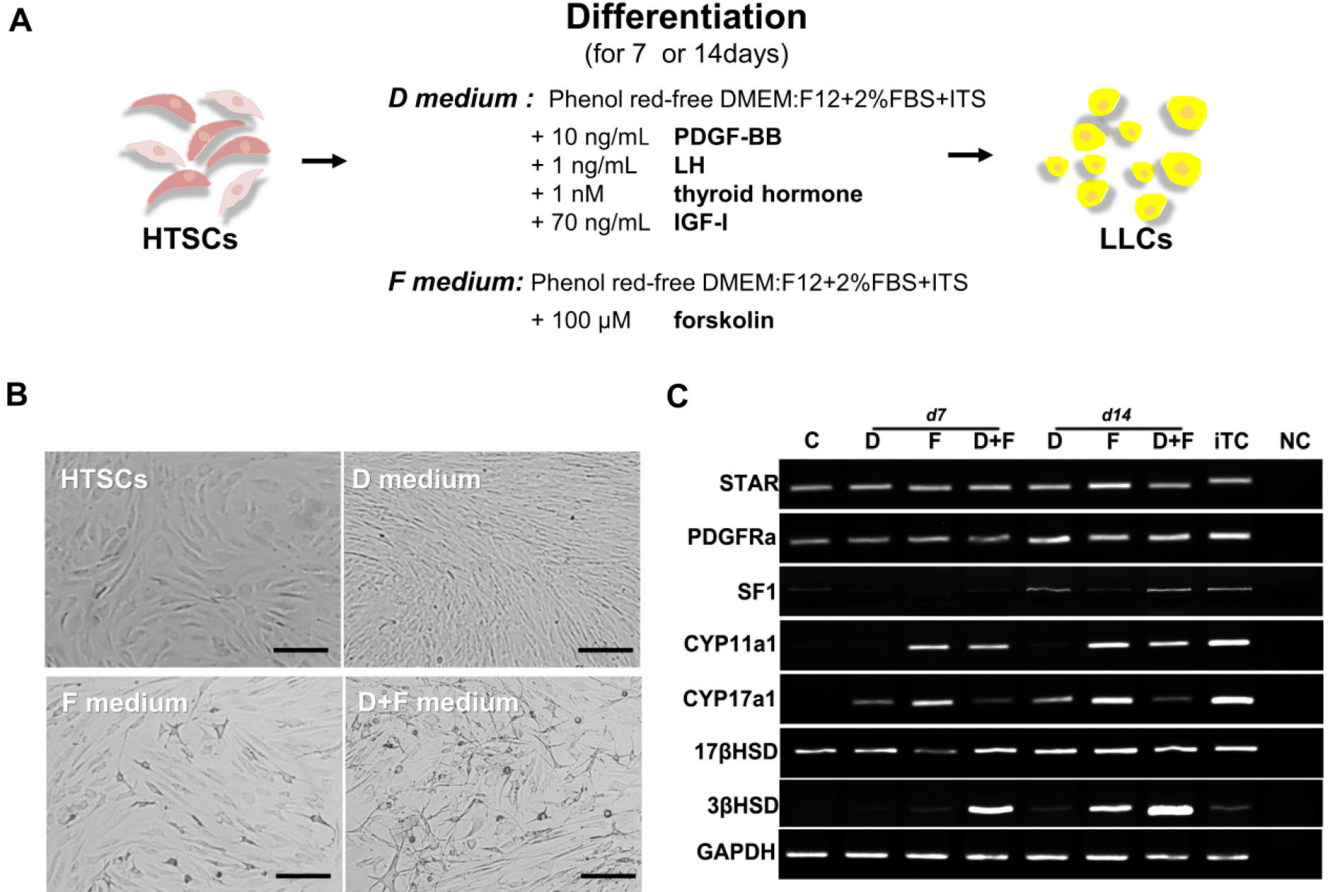
Differentiation of HTSCs into LLCs. **A** Schematic strategy for the induction of LLCs from HTSCs. **B** Representative images of HTSCs and HTSC-derived LLCs from three different methods over 14 days. Scale bars = 200 μm. **C** mRNA expression levels of markers for steroidogenic cells (STAR and SF1) and Leydig cells (PDGFRα, CYP11a1, CYP17a1, 17βHSD, and 3βHSD) in induced LLCs derived from HTSCs. **C** HTSCs as control cells; iTC, isolated testicular cell; NC, negative control; HTSCs, highly proliferative testis-derived stem cells; LLCs, Leydig cell-like cells; STAR, steroidogenic acute regulatory protein; SF1, steroidogenic factor 1; PDGFRα, platelet-derived growth factor receptor-alpha; CYP11a1, cytochrome P450 cholesterol side-chain cleavage enzyme; CYP17a1, cytochrome P450 17α-hydroxylase; 17βHSD, 17β-hydroxysteroid dehydrogenase; 3βHSD, 3β-hydroxysteroid dehydrogenase; GAPDH, glyceraldehyde 3-phosphate dehydrogenase; DMEM, Dulbecco’s modified Eagle’s medium; FBS, fetal bovine serum; ITS, insulin-transferrin-selenium

**Fig. 4:**
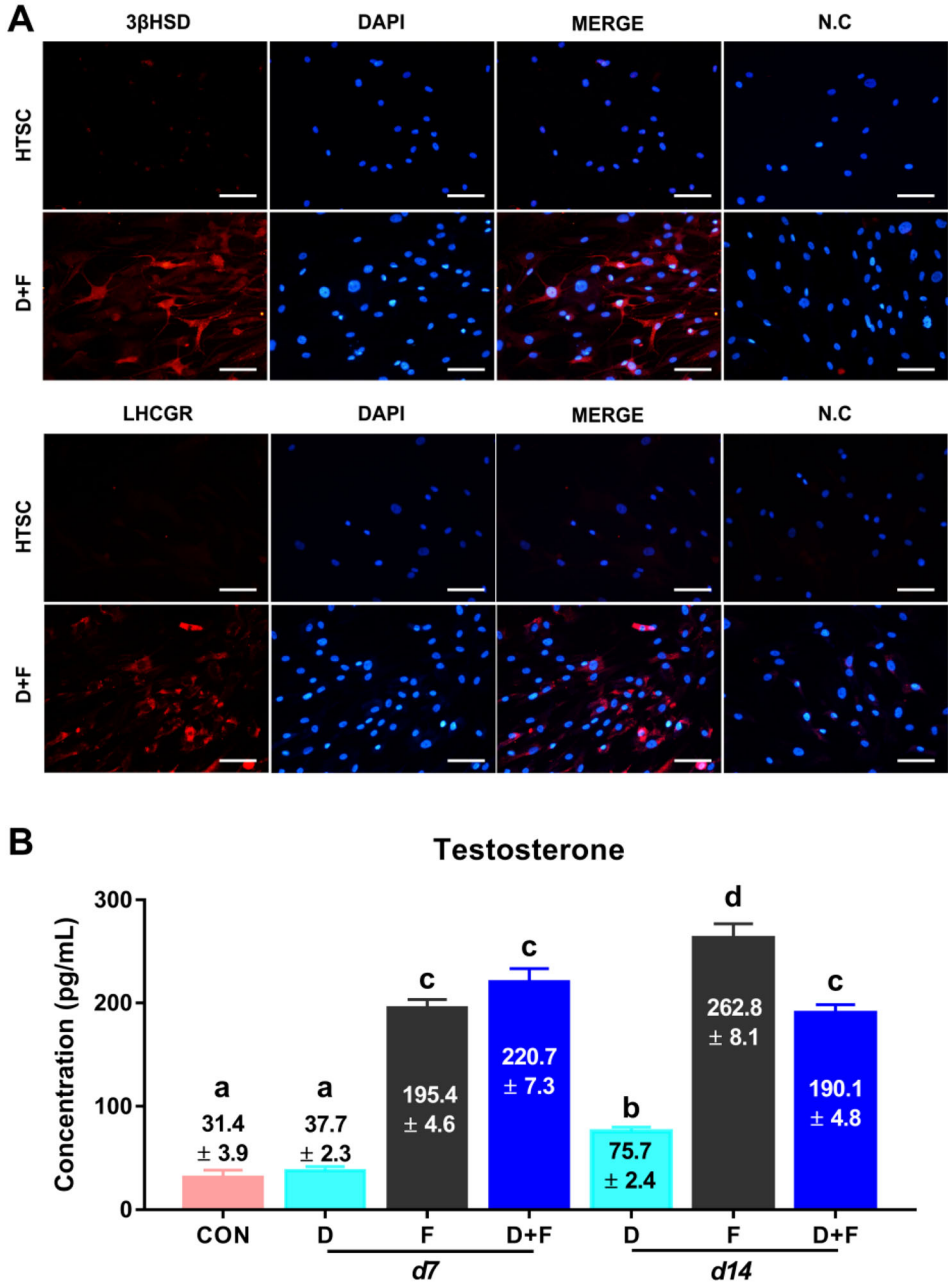
Characterization of HTSCs-derived LLCs. **A** Immunofluorescence staining of markers for Leydig cells (3βHSD and LHCGR) in LLCs differentiated from HTSCs. Scale bars = 100 μm. **B** Medium testosterone levels in different groups, as determined by ELISA. The supernatant of HTSCs was used as the negative control (CON). Data are presented as the mean ± standard error (SE, n = 3). Different superscripts designate significant differences (*p* < 0.05); N.C, negative control; HTSCs, highly proliferative testis-derived stem cells; LLCs, Leydig cell-like cells; 3βHSD, 3β-hydroxysteroid dehydrogenase; LHCGR, luteinizing hormone and choriogonadotropin receptor

To investigate whether HTSCs can differentiate into LCs *in vitro* and determine the optimal conditions for LC differentiation, cells were treated with three different medium conditions (Fig. 3A). D medium (consisting of PBGF-BB, LH, thyroid hormone, and IGF-1) was previously reported by Ge et al. [31]. D medium is widely used to induce the differentiation of SLCs into LCs [5, 18, 19, 32–35]. F medium contains forskolin (FSK), which is a stimulator of adenylate cyclase that results in increased adenosine-3′,5-cyclic monophosphate (cAMP) production [36]. According to previous studies, FSK can induce the differentiation of SLCs and mouse ES into LLCs [20, 32]. D + F medium is a mixture of D and F media. All types of medium induced morphological changes in HTSCs (Fig. 3B). We then analyzed the expression of ALC markers STAR, platelet-derived growth factor receptor-alpha (PDGFRα), SF1, CYP11a1, CYP17a1, 17βHSD, and 3βHSD after treatment with D and F media. Media D and F induced the expression of ALC markers on day 14 after differentiation, but their expressions were weak on day 7. Since the purpose of this study was to develop a fast and efficient differentiation method for the generation of LLCs, to enhance the efficiency, cells were treated with a mixture of media D and F. As a result, D + F medium induced the expression of all markers within the shortest period (Fig. 3C). In particular, the expression of 3βHSD, an enzyme that acts in the final stage of testosterone synthesis and is an important marker of ALCs, was highest in the D + F group on both days 7 and 14 after differentiation. To further characterize LLCs, the protein expression of 3βHSD and LHCGR was confirmed by immunocytochemistry, which showed a strong expression of both proteins, but HTSCs were negative for these proteins (Fig. 4A). Testosterone can be produced from all groups of LLCs on day 14 after differentiation, and it was significantly higher than that in the control (HTSCs) group. However, on day 7 after treatment, D + F medium had the highest levels of testosterone compared to the other groups (Fig. 4B). Taken together, these results indicate that HTSCs possess SLC properties and can generate functional LLCs in D + F medium. Therefore, this is a much more rapid and efficient method to induce the differentiation of HTSCs into LLCs.

### Generation of IM from hESCs

**Fig. 5:**
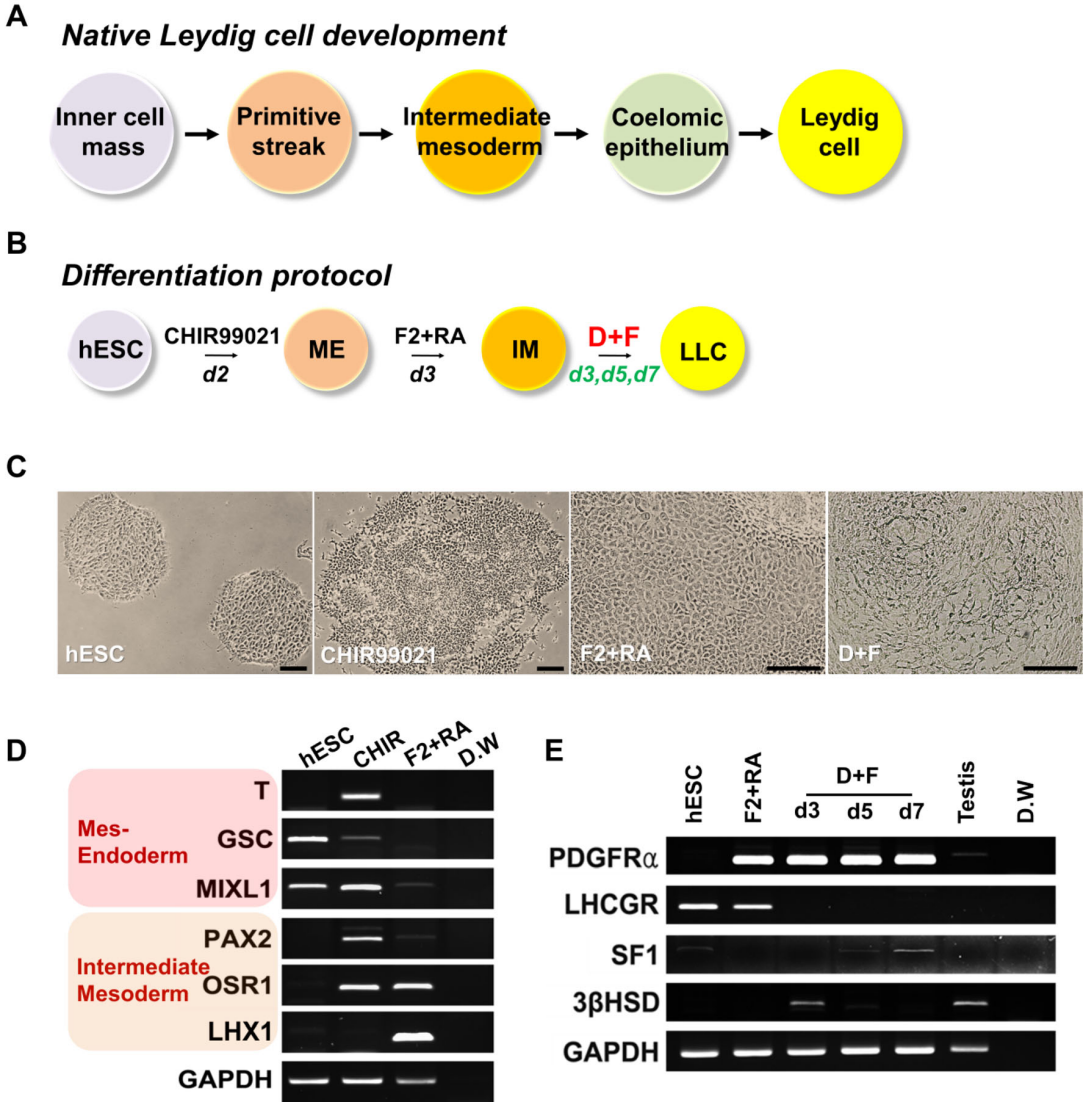
Induction of hESCs into ME, IM and LLCs. The D + F medium is insufficient to generate LLCs from hESCs. **A** Diagram of developmental stages of native Leydig cells during embryogenesis. **B** Schematic of directed differentiation of hESCs into LLCs through the ME and IM. The derivation methods are presented. **C** Representative images of hESCs, ME induced by CHIR99021, IM induced by F2 + RA, and LLCs induced by D + F medium. Scale bars = 200 μm. **D** mRNA expression levels of markers for the ME (T, GSC, and MIXL1) and IM (PAX2, OSR1, and LHX) in hESCs, ME, and IM. **E** Gene expression of Leydig cell markers in hESC-derived LLCs treated with D + F medium for 3, 5, or 7 days. ME, mesendoderm; IM, intermediate mesoderm; LLCs, Leydig cell-like cells; F2, FGF2; RA, retinoic acid; T, Brachyury; GSC, goosecoid; MIXL1, Mix-like 1; PAX2, Paired box 2; OSR1, odd-skipped related 1; LHX, LIM homeobox-1; PDGFRα, platelet-derived growth factor receptor-alpha; LHCGR, luteinizing hormone and choriogonadotropin receptor; SF1, steroidogenic factor 1; 3βHSD, 3β-hydroxysteroid dehydrogenase; GAPDH, glyceraldehyde 3-phosphate dehydrogenase

Although it is unclear where the LCs originate from early embryonic tissues, most studies have suggested that they probably arise from the coelomic epithelium, gonadal mesonephric border region, and mesoderm [29, 37, 38]. The IM is a part of the mesoderm, which is located between the lateral plate mesoderm and paraxial mesoderm of three germ layers and can generate the kidney, gonads, and genital tracts (Fig. 5A) [39, 40]. Therefore, we assumed that IM cells might differentiate into LLCs. Furthermore, our previous data showed that Sertoli-like cells, which have a similar origin to LCs, can be successfully generated from the IM [41]. Thus, we applied this strategy to generate LLCs from hESCs. Based on the embryological sequence of LC differentiation, we defined a 3-stage for the differentiation of hESCs into LLCs (Fig. 5B).

Lam et al. demonstrated that the IM can be efficiently generated from the mesendoderm; therefore, the first step was the induction of the mesendoderm (ME) from hESCs by CHIR99021, which is known as glycogen synthase kinase-3β (GSK-3β) inhibitor, for 2 days [42]. Consistent with their report, CHIR99021-treated cells showed epithelial to mesenchymal transition, migrating away from the margin of hESC colonies (Fig. 5C). Furthermore, the expression of ME markers (T, GSC, and MIXL1) was observed in these cells, suggesting that ME was successfully generated by CHIR99021 treatment (Fig. 5D). Next, to induce the IM from ME, hESC-derived ME was treated with FGF2 and RA for 3 days. FGF2-and RA-treated MEs proliferated and expressed IM markers (PAX2, OSR1, and LHX1; Fig. 5C, D). These results suggest that ME differentiated into the IM.

### Generation of LLCs from hESC-derived IM

As mentioned above, D + F medium can induce the differentiation of LLCs, which secrete more testosterone in a shorter time than other conditions, from HTSCs, which might serve as progenitor cells for LCs. Thus, to generate LLCs from IM, we treated the IM with D + F medium. Because this study aimed to develop a method for the rapid generation of LLCs from hESCs, we tested various durations of treatment and evaluated the expression of LC markers on days 3, 5, and 7 of differentiation. Interestingly, IM cells expressed PDGFRα, which is a putative marker for SLCs [31, 32], suggesting that IM might already be primed to differentiate into the LC lineage (Fig. 5E). 3βHSD, which is an LC marker, was expressed on day 3 of differentiation, but its expression disappeared as time progressed. In contrast, SF1, which is a steroidogenic marker, was expressed from day 5 of D + F treatment, and it was well expressed over time. However, LHCGR was not expressed, regardless of the treatment period. Therefore, the duration of the differentiation medium treatment for LLC differentiation was fixed to this intermediate period, i.e. on day 4 of treatment, but we speculated that additional factors might be needed to induce the expression of all markers.

**Fig. 6:**
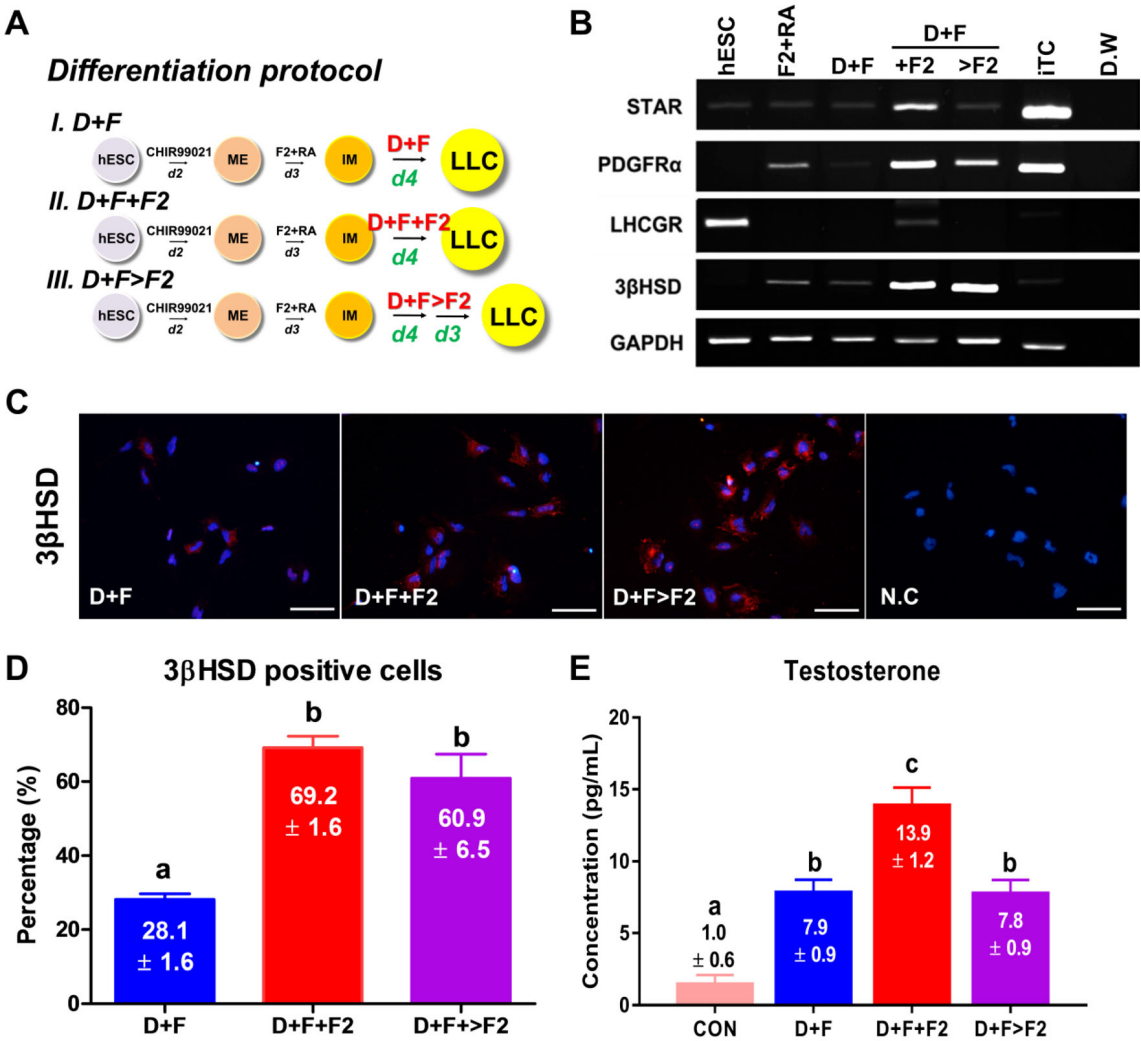
Characterization and differentiation of hESCs into LLCs. **A** Schematic of different methods of inducing the differentiation of LLCs from the IM. **B** Gene expression of Leydig cell markers in hESC-derived LLCs treated with D + F, D + F + F2, or D + F > F2. **C** Immunofluorescence staining of 3βHSD in LLCs differentiated from hESCs. Scale bars = 100 μm. **D** Quantification of 3βHSD-positive cells in each group. The percentage of 3βHSD-positive cells was determined. Three sections per slide were counted. **E** Medium testosterone levels in different groups, as determined by ELISA. The supernatant of hESCs was used as the negative control (CON). Data are expressed as the mean ± standard error (SE, n = 3). Different superscripts indicate significant differences (*p* < 0.05). N.C, negative control; hESCs, human embryonic stem cells; LLCs, Leydig cell-like cells; IM, intermediate mesoderm; 3βHSD, 3β-hydroxysteroid dehydrogenase

Liu et al. demonstrated that FGF2 can promote SLC proliferation and differentiation of SLCs into testosterone-producing LCs [43]. To optimize the efficiency of inducing LLCs, at the final induction stage, we added FGF2 to D + F medium (D + F + F2) or after treatment with D + F medium (D + F > F2; Fig. 6A). The D + F + F2 and D + F > F2 groups showed obvious expression of PDGFRa and 3βHSD, but they were indistinct in the D + F groups. In particular, STAR and LHCGR were more strongly expressed only in the D + F + F2 group (Fig. 6B). To further evaluate the optimal conditions, the protein expression of 3βHSD was observed using an immunofluorescence assay (Fig. 6C). Statistical data on the percentages of these cells are shown in Fig. 6D. The results showed that the FGF2-supplemented group had significantly more 3βHSD positive cells than the D + F group. These results are consistent with that of RT-PCR. Finally, we measured the levels of testosterone secreted by the LLCs from each group. Results of ELISA showed that LLCs from the D + F + F2 group produced significantly higher levels of testosterone than those from the other groups. Taken together, these results demonstrate that D + F + F2 is a more efficient system than the others for the induction of LLCs.

## Discussion

The life expectancy of humans has increased due to advances in medicine; thus, the desire to maintain vitality and improve the quality of life after aging is increasing. In males, testosterone is an essential hormone that regulates the physiology of the whole body [44]. Testosterone dysfunction can occur due to aging, trauma, radiotherapy, or chemotherapy, which can result in various diseases, such as sexual dysfunction, cardiovascular disease, and osteoporosis, and is known to affect approximately 38.7% of males over the age of 45 [6, 45]. Although TRT has been used to overcome testosterone dysfunction, it is not fully capable of restoring the physiological patterns of testosterone metabolism. Therefore, it is necessary to develop an alternative method to TRT to mimic the physiological patterns of testosterone in hypogonadism.

LCs, which produce a vast majority of testosterone in males, are a promising cell source for the treatment of male hypogonadism. However, it is difficult to secure enough cell count for treatment because LCs have limited proliferation capacity and are rare in the testis. To overcome this, we generated LLCs from hESCs, which have an unlimited proliferation potential. Recently, SLCs, which are progenitor stem cells for LCs, have been utilized to understand LC development and for the treatment of hypogonadism [6]. We speculated that if we devise a proper strategy for generating LLCs from SLCs, we can develop an efficient method for the differentiation of hESCs into LLCs.

**Fig. 7:**
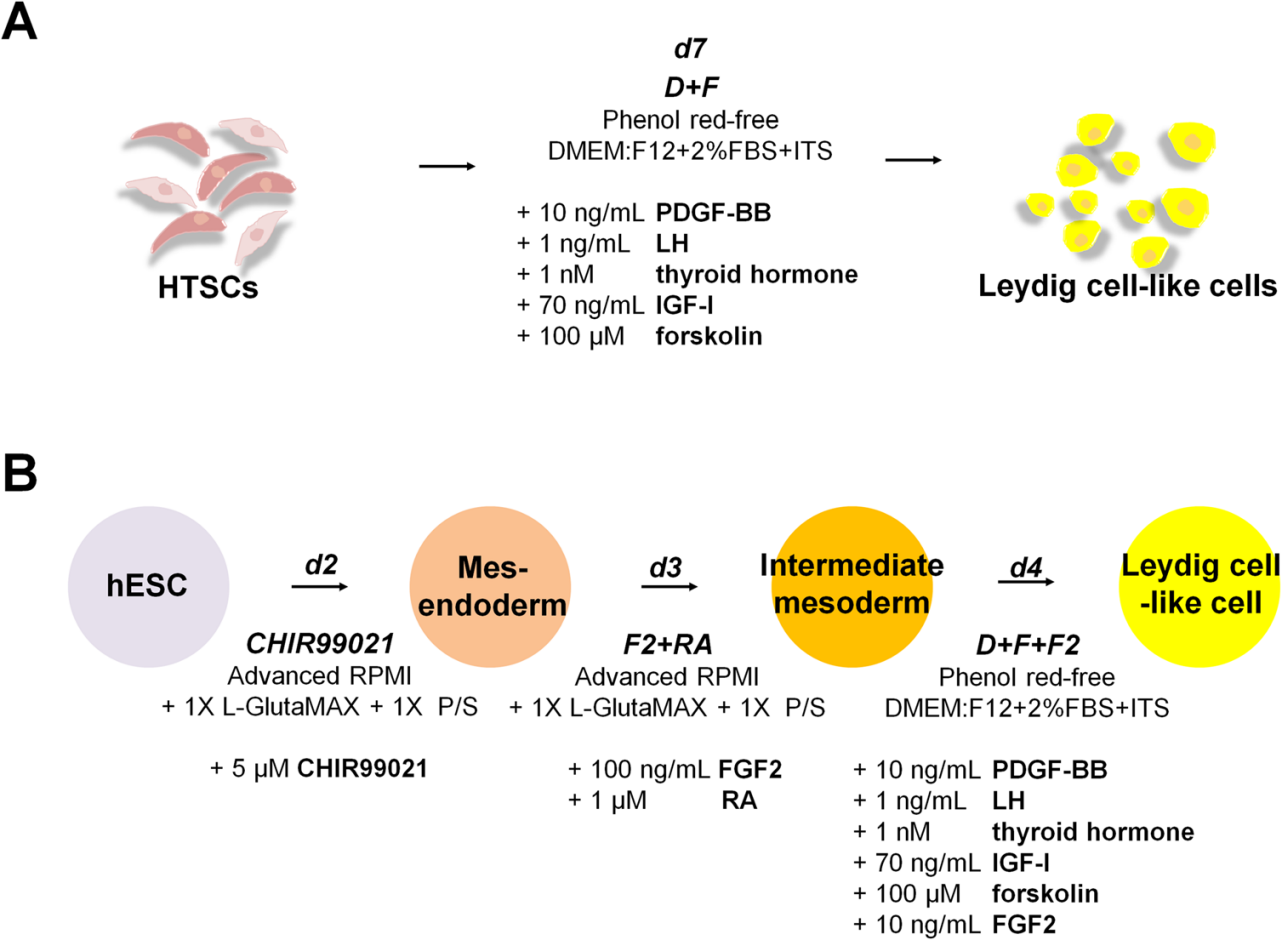
Schematic illustration of the protocol for LLCs differentiation from HTSCs and hESCs. **A** Diagram showing the differentiation of HTSCs into LLCs. HTSCs were cultured in DMEM/F12 supplemented with 2% FBS and ITS and exposed to D + F medium, containing PDGF-BB, LH, thyroid hormone, IGF-I, and forskolin, for 7 days to form LLCs. **B** Diagram showing the stepwise differentiation of hESCs into LLCs. A day before ME induction, hESCs were plated on Geltrex-coated plates and cultured in hESC medium for attachment. On induction day 0, cells were cultured in CHIR medium for 2 days to induce the formation of the ME. Then, cells were incubated in F2 + RA medium for 3 days to induce the formation of the IM. To further specify LLCs, cells were treated with D + F + F2 medium containing PDGF-BB, LH, thyroid hormone, IGF-I, forskolin, FGF2 for 4 days on the IM. On induction day 9, LLCs were formed. HTSCs, highly proliferative testis-derived stem cells; hESCs, human embryonic stem cells; LLCs, Leydig cell-like cells; IM, intermediate mesoderm; ME, mesendoderm; ITS, insulin-transferrin-selenium (ITS); DMEM, Dulbecco’s modified Eagle’s medium; FBS, fetal bovine serum; ITS, insulin-transferrin-selenium

In the present study, we identified that HTSCs possessed SLCs properties and could be efficiently differentiated into LLCs in the D + F medium, which consisted of PDGF-BB, LH, thyroid hormone, IGF-I, and forskolin, within 7 days (Figs. 2,3,4, 7A). All conditions could induce the production of testosterone-secreting LLCs from HTSCs; thus, it was confirmed that HTSCs are a valuable cell source for LC transplantation therapy. Furthermore, it might be possible to use autologous SLC transplantation. In summary, in the present and previous studies, we confirmed that HTSCs have not only high proliferative ability but also differentiation ability to various cell types, such as the three germ layer lineages and LCs [17].

Despite these advantages, it is still necessary to develop universally usable LCs for stable clinical applications. In this study, we proposed a rapid and efficient system for the differentiation of hESCs into LLCs based on embryonic development *in vivo*. Although several studies have reported that LCs can be generated from various stem cells, most studies require the additional expression of ectopic genes and longer time [6, 20–22, 24–28]. To overcome these issues, we developed a direct method of inducing LCs from hESCs using a few defined molecular compounds within 9 days.

LCs and SLCs originate from the coelomic epithelium and mesonephros formed by the intermediate mesoderm. Lam et al. demonstrated that sequential treatment with CHIR99021, FGF2, and RA can efficiently generate IM from hESCs through the ME stage [42]. We established a three-step differentiation strategy to mimic these embryological processes *in vitro*. In the first stage, we induced the differentiation of the ME from hESCs using CHIR99021 and then treated the cells with FGF2 and RA to induce differentiation into the IM. Finally, LLCs were generated from the IM by treatment with D + F + F2 medium composed of PDGF-BB, LH, thyroid hormone, IGF-I, forskolin, and FGF2 (Fig. 7B). Unlike HTSCs, induction of LLCs from the IM is needed for additional FGF2 supplementation. This is probably because the IM is formed at a much earlier stage or is not a defined LC lineage like SLCs. Nonetheless, this medium can generate LLCs from the hESC-derived IM within 4 days after treatment, which is very beneficial in terms of reducing the cost of preparing cells for clinical application.

In conclusion, our present study demonstrated that HTSCs might be putative SLCs in the testis and presented the development of a safe, rapid, and efficient strategy for the direct differentiation of hESCs into LLCs. These findings could contribute to a safer and more cost-effective strategy for further clinical use of LC transplantation therapy for male hypogonadism.
